# Interactive Effects of Dietary Guanidinoacetic Acid and Energy Density on Growth Performance, Carcass Characteristics, Meat Quality, and Heat Stress Tolerance in Broilers

**DOI:** 10.3390/life16081225

**Published:** 2026-07-24

**Authors:** Noman Ahmed, Muhammad Yousaf, Muhammad Muneeb, Chaudhry Ahsan Akram, Irfan Ahmed, Muhammad Qumar, Abdul Ghayas, Muhammad Asif, Ali R. Al Sulaiman, Ala E. Abudabos

**Affiliations:** 1 Department of Animal Nutrition, Faculty of Veterinary and Animal Sciences, The Islamia University of Bahawalpur, Punjab 63100, Pakistan; nomannil82@gmail.com (N.A.);; 2 Institute of Feed Research, Chinese Academy of Agricultural Sciences, Beijing 100193, China; muneebsaqib5826@gmail.com; 3 Department of Livestock and Poultry Production, Faculty of Veterinary Sciences, Bahauddin Zakariya University, Multan 60800, Pakistan; 4 Department of Poultry Science, Faculty of Veterinary and Animal Sciences, The Islamia University of Bahawalpur, Punjab 63100, Pakistan; 5 Ajwa Feeds, Multan 60000, Pakistan; 6 Environmental Protection Technologies Institute, Sustainability and Environment Sector, King Abdulaziz City for Science and Technology, P.O. Box 6086, Riyadh 11442, Saudi Arabia; arsuliman@kacst.gov.sa; 7 Department of Food and Animal Sciences, College of Agriculture, Tennessee State University, Nashville, TN 37209, USA

**Keywords:** guanidinoacetic acid, broiler, growth performance, meat quality, energy sparing

## Abstract

Guanidinoacetic acid (GAA), a natural precursor of creatine, plays a pivotal role in cellular energy metabolism. This study investigated the effects of dietary GAA supplementation at varying levels (0, 0.6, and 1.2 g/kg) combined with standard or reduced (−100 kcal/kg) dietary energy on growth performance, carcass characteristics, meat quality, and heat stress tolerance (during the finisher phase) in broiler chickens. A total of 360 one-day-old male Ross-308 broiler chicks were randomly allocated to 36 floor pens in a 3 × 2 factorial arrangement under a completely randomized design. Six treatments with six replicates, each having 10 birds, were evaluated over 35 days. Growth performance parameters were recorded weekly. On day 35, samples were collected for carcass characteristics (dressing percentage, breast and thigh yield, and organ weights), meat quality analysis (drip loss, cooking loss, and color parameters), and heat stress response. The heat stress response (panting frequency) was recorded during the finisher phase of the trial. GAA supplementation at 1.2 g/kg significantly (*p* < 0.001) improved final body weight, total weight gain, feed conversion ratio, dressing percentage, breast yield, and thigh yield. Meat quality parameters showed significant improvement (*p* < 0.001) with GAA in a dose-dependent manner for drip loss, cooking loss, and all color parameters (L*; a*; b*). Significant GAA × energy interactions (*p* < 0.001) were observed for final body weight, total weight gain, feed intake, FCR, cooking loss, live body weight at slaughter, breast yield, liver weight, gizzard weight, intestine weight, and panting frequency. The effect of GAA on heat stress tolerance was context-dependent, with a significant GAA × energy interaction (*p* < 0.001) for panting frequency. Under reduced-energy conditions, 1.2 g/kg GAA decreased panting frequency, suggesting improved thermoregulatory capacity, while under standard-energy conditions, GAA increased panting frequency. The main effect of GAA on panting frequency was not significant (*p* = 0.38), while the main effect of energy was highly significant (*p* < 0.001). In conclusion, GAA supplementation at 1.2 g/kg significantly improves growth performance, feed efficiency, meat quality (reduced drip and cooking losses and improved color parameters), and carcass yield in broiler chickens. Importantly, GAA supplementation largely compensated for a 100 kcal/kg reduction in dietary energy while maintaining performance. These findings support GAA as a promising nutritional strategy for sustainable broiler production, particularly in hot-climate regions.

## 1. Introduction

The global poultry industry plays a vital role in meeting the increasing demand for affordable, high-quality animal protein. Broiler production, in particular, has intensified significantly to achieve rapid growth rates and superior feed efficiency. However, this genetic progress has placed substantial metabolic demands on modern broiler chickens, making them highly susceptible to nutritional imbalances, metabolic disorders, and environmental stressors such as heat stress [1].

Energy is the primary limiting nutrient for growth, and cellular energy metabolism relies heavily on adenosine triphosphate to sustain various physiological processes [2]. Guanidinoacetic acid (GAA; N-(aminoimino-methyl)-glycine, also known as glycocyamine) is a naturally occurring amino acid derivative and the immediate precursor of creatine, a key molecule in cellular energy homeostasis [3]. Creatine is phosphorylated to form phosphocreatine, which acts as an energy buffer in high-demand tissues such as skeletal muscle, the heart, and the brain. The phosphocreatine/creatine kinase system rapidly regenerates adenosine triphosphate from adenosine diphosphate, thereby maintaining cellular energy charge during periods of high energy demand [4].

GAA is endogenously synthesized in the avian kidney and liver through a reaction catalyzed by L-arginine amidinotransferase, utilizing arginine and glycine as substrates [5]. Subsequently, GAA is methylated by S-adenosyl methionine via guanidinoacetate N-methyltransferase in the liver to produce creatine [6]. Endogenous synthesis of creatine, however, may be insufficient to meet the demands of fast-growing modern broiler strains [7]. Dietary GAA supplementation has emerged as an effective nutritional strategy to enhance creatine availability and improve energy metabolism in poultry [8]. GAA exhibits superior thermal stability compared to creatine, making it highly suitable for feed manufacturing processes involving high temperatures and pressure [6]. Several studies have demonstrated that GAA supplementation improves growth performance, feed conversion ratio (FCR), and breast meat yield in broiler chickens [9,10]. Furthermore, GAA has been shown to possess energy-sparing properties, allowing for dietary energy reduction without compromising productivity [11,12]. Tossenberger et al. [6] reported complete digestibility of supplemental GAA in broilers, with negligible endogenous losses, confirming its high bioavailability.

Heat stress remains a major constraint to broiler production in tropical and subtropical regions, causing reduced feed intake, impaired growth, increased mortality, and compromised meat quality [13]. Heat-stressed birds exhibit elevated panting frequency as an adaptive thermoregulatory response, which increases metabolic heat production and energy expenditure [1]. Emerging evidence suggests that GAA supplementation may mitigate the adverse effects of heat stress by enhancing cellular energy status, improving antioxidant capacity, and modulating the stress response [14,15]. However, the interactive effects of GAA and dietary energy levels on heat stress tolerance in broilers require further investigation.

The quality of poultry meat is increasingly important to consumers, with water-holding capacity (WHC), color, and tenderness being key determinants of acceptability and economic value [16]. Post mortem pH decline and protein denaturation significantly influence WHC, and interventions that stabilize cellular energy status pre-slaughter may improve meat quality [17]. GAA supplementation has been shown to reduce drip loss and cooking loss while improving meat redness, indicating enhanced oxidative stability and myoglobin content [17,18]. Nonetheless, dose–response relationships and interactions with dietary energy levels remain incompletely characterized. Despite substantial evidence supporting GAA efficacy, knowledge gaps persist regarding optimal inclusion levels under different dietary energy regimens, particularly in heat-stressed environments. Therefore, this study was conducted to determine the effects of dietary GAA supplementation at 0, 0.6, and 1.2 g/kg combined with standard versus reduced (−100 kcal/kg) dietary energy levels on (1) growth performance parameters, (2) carcass characteristics and organ weights, (3) meat quality attributes (drip loss, cooking loss, and color), and (4) physiological response to acute heat stress in Ross 308 broiler chickens. Based on the aforementioned scientific rationale, it was hypothesized that dietary supplementation with GAA would enhance growth performance, improve carcass characteristics, and positively modulate meat quality parameters and heat tolerance in broilers. The −100 kcal/kg energy reduction was selected based on previous studies demonstrating that broiler diets can be reduced by up to 100–150 kcal/kg without severely compromising performance when supplemented with appropriate feed additives [12,19]. This level of reduction represents a practically feasible energy saving (approximately 3–5% of dietary energy) that has significant economic implications in commercial broiler production, where feed costs account for 70–75% of total production costs. The GAA doses (0.6 and 1.2 g/kg) were selected based on previously reported effective ranges in broiler diets [8,9], with the higher dose (1.2 g/kg) expected to provide sufficient creatine precursor to potentially compensate for the energy reduction, while the lower dose (0.6 g/kg) served as a mid-range reference based on commercial recommendations.

This study extends previous research by systematically investigating the interactive effects of graded GAA levels (0, 0.6, and 1.2 g/kg) with standard versus reduced (−100 kcal/kg) dietary energy levels in a comprehensive 3 × 2 factorial design. While previous studies have individually examined GAA’s effects on growth performance [8,9], meat quality [17,18], or heat stress responses [14,15], to our knowledge, this is the first study to simultaneously evaluate all these parameters under both standard- and reduced-energy conditions in a single experimental framework. Furthermore, the novel finding of a significant GAA × energy interaction for heat stress responses, with context-dependent effects on panting frequency, provides new insights into the complex relationship between energy metabolism and thermoregulation that have not been previously reported.

## 2. Materials and Methods

The study was conducted with the approval of the Advanced Study and Research Board, Islamia University of Bahawalpur, Pakistan (approval no.: AN1/2026/464).

## 3. Experimental Design

In total, 360 male day-old Ross-308 broiler chicks were procured from a local hatchery. Birds were weighed individually and allocated to floor pens using a completely randomized design. The experiment consisted of six dietary treatments (*n* = 60 chicks per treatment) having six replicates of 10 birds each. The six dietary treatments were as follows: (1) basal diet with standard energy and no GAA; (2) diet with reduced energy, −100 kcal/kg, and no GAA; (3) standard-energy diet supplemented with 0.6 g/kg GAA; (4) reduced-energy diet, −100 kcal/kg, supplemented with 0.6 g/kg GAA; (5) standard-energy diet supplemented with 1.2 g/kg GAA; and (6) reduced-energy diet, −100 kcal/kg, supplemented with 1.2 g/kg GAA (Table 1).

GAA was provided through the feed additive GuanAMINO^®^ (96% GAA; Evonik Animal Nutrition, Essen, Germany).

The birds were reared in accordance with the management standards outlined in the Ross broiler management handbook [20]. The diets were designed according to the feeding standards of Ross broiler nutrition specifications [21] for the starter, grower, and finisher phases. Table 2 presents the composition and nutritional contents of the basal diets.

## 4. Parameters Evaluated

Body weight (BW) and feed intake (FI) were measured weekly, and measurements were averaged for each pen. The FCR adjusted for mortality, was determined for each pen.

On day 35, two birds per replicate (12 per treatment) were randomly selected, weighed, and slaughtered. Breast meat samples were collected for analysis, and meat color (CIE L*, a*, and b*) was measured 15 min post-slaughter using a Chroma meter CR-410 (Konica Minolta Sensing, Inc., Osaka, Japan) according to the meat color measurement guidelines [22]. Drip loss was determined using standard protocol by suspending meat samples of approximately 50 g in sealed containers at 4 °C for 24 h and calculating weight loss as [(initial weight − final weight)/initial weight] × 100. Cooking loss was determined according to standard procedure [22], by vacuum-sealing samples, cooking them in a water bath to an internal temperature of 75 °C, and calculating cooking loss as [(raw weight − cooked weight)/raw weight] × 100.

After feather removal and evisceration, carcasses were weighed, and dressing percentage was calculated as [(carcass weight/live weight) × 100]. Breast and thigh muscles were then separated and weighed, and the liver, gizzard, and intestines were removed and weighed. All tissue and organ weights were expressed as percentages of live body weight. For all parameters measured, the pen served as the experimental unit. For carcass characteristics, meat quality, and panting frequency, two birds per pen were randomly selected and measured, and the average of these two birds was used as the replicate value for each pen. This approach ensured that pseudoreplication was avoided and the pen remained the true experimental unit.

Cyclic heat stress was applied from 27 to 35 days of age using a cyclic heat exposure protocol, in which birds were exposed to 34 °C for 8 h during the day (08:00–16:00) and returned to thermoneutral conditions of approximately 22–24 °C for the remainder of the day (16:00–08:00). The system was programmed to maintain 34 °C ± 1 °C during the heat exposure period (8 h/day) and 22–24 °C ± 1 °C during the thermoneutral period, with relative humidity maintained at 55–65% throughout the trial. On day 35, panting frequency, expressed as breaths per minute, was measured 4 h after the onset of heat stress in two randomly selected birds per pen [23]. Temperature was controlled using a computerized environmental control system (TempTron T-607; AgroLogic Ltd., Netanya, Israel) equipped with thermostatically controlled heaters, evaporative cooling pads, and exhaust fans. Temperature was monitored continuously using digital data loggers (Poulta, Inc., Newark, DE, USA).

## 5. Statistical Analysis

The collected data were first checked for normality and homogeneity of variance. The normality and homogeneity of variance of the data were assessed using the Kolmogorov–Smirnov test and the Levene test, respectively. Subsequently, the data were analyzed using a two-way ANOVA with the General Linear Model in Minitab 17 statistical software (Minitab, LLC, State College, PA, USA), with GAA level and energy level included as fixed factors. The pen served as the experimental unit. All data are presented as least-squares means with pooled standard error of the mean (SEM), and superscript letters indicate significant differences between treatment groups (*p* < 0.05; Tukey’s HSD post hoc test).

## 6. Results

### 6.1. Growth Performance

Significant effects (*p* < 0.001) of both GAA and dietary energy level were observed for all growth performance parameters. GAA supplementation exhibited a significant (*p* < 0.001) response across final body weight, weight gain, feed intake, and FCR (Table 3). Birds receiving the highest level of GAA (1.2 g/kg) demonstrated the greatest final body weight (2144.50 g), which was significantly higher than both the 0.6 g/kg GAA group (2081.50 g) and the control group without GAA (2006.67 g; *p* < 0.001). Weight gain followed the same pattern, with the 1.2 g/kg GAA group achieving 2101.67 g compared to 2038.50 g in the 0.6 g/kg group and 1963.50 g in the control group (*p* < 0.001). Feed intake was significantly influenced by GAA supplementation (*p* < 0.001), with birds fed 0.6 g/kg GAA consuming the most feed (3018.83 g), while the control group had the lowest intake (2974.33 g). The most pronounced effect of GAA was observed on FCR, where supplementation with 1.2 g/kg GAA resulted in the most efficient FCR (1.43), which was significantly better than the 0.6 g/kg GAA group (1.48) and the control group (1.51; *p* < 0.001). Energy reduction impaired all growth parameters (*p* < 0.001). For final body weight, the significant GAA × energy interaction (*p* < 0.001) indicated that the effect of GAA depended on energy level. Under standard-energy conditions, 1.2 g/kg GAA increased final body weight compared to the control (2155.59 vs. 2050.55 g; *p* < 0.05). Similarly, under reduced-energy conditions, 1.2 g/kg GAA increased final body weight compared to the control (2134.53 vs. 1962.50 g; *p* < 0.05). This indicates that 1.2 g/kg GAA largely compensated for the energy reduction, as birds achieved comparable body weights to those fed standard-energy diets with GAA supplementation. Similarly, the significant interaction observed for FCR (*p* < 0.001) showed that both 1.2 g/kg GAA groups, under standard- and reduced-energy conditions, achieved the best feed efficiency, with FCR values of 1.42 and 1.43, respectively, significantly outperforming all other treatment groups.

### 6.2. Meat Quality

GAA supplementation significantly improved WHC in a dose-dependent manner (*p* < 0.001) (Table 4). Drip loss decreased progressively with increasing GAA levels: control birds had the highest drip loss (3.75%), followed by 0.6 g/kg GAA (2.98%), and the lowest was observed in the 1.2 g/kg GAA group (2.14%; *p* < 0.001). Similarly, cooking loss was significantly reduced by GAA supplementation (*p* < 0.001), with the control group having the highest cooking loss (23.82%), followed by 0.6 g/kg GAA (22.12%), and the 1.2 g/kg GAA group having the lowest (19.99%), representing a 16.1% improvement in cooking yield. Regarding meat color, GAA supplementation significantly modified all parameters (L*, a*, and b*) (*p* < 0.001). Energy reduction negatively impacted drip loss, cooking loss, and meat color (*p* < 0.001). A significant GAA × energy interaction was observed for cooking loss (*p* < 0.001), indicating that the effect of GAA on cooking loss depended on the dietary energy level.

### 6.3. Carcass Characteristics

GAA supplementation significantly increased live body weight at slaughter in a dose-dependent manner (*p* < 0.001) (Table 5). Dressing percentage followed the same pattern, with the 1.2 g/kg GAA group having the highest dressing percentage (74.89%), compared to 73.07% for 0.6 g/kg GAA and 71.19% for the control (*p* < 0.001). Dietary energy reduction significantly decreased both live body weight (2001.23 vs. 2070.61 g; *p* < 0.001) and dressing percentage (72.18 vs. 73.92%; *p* < 0.001). A significant GAA × energy interaction was observed for live body weight (*p* < 0.001), revealing that the 1.2 g/kg GAA groups performed best regardless of energy level, with both the standard energy (2112.13 g) and reduced energy (2099.12 g) groups being statistically similar and superior to all other treatments. For dressing percentage, the interaction was not significant (*p* = 0.15).

GAA supplementation significantly increased both breast and thigh meat yields in a dose-dependent manner (*p* < 0.001). Dietary energy reduction significantly decreased both breast yield (22.09 vs. 24.06%; *p* < 0.001) and thigh yield (17.45 vs. 18.07%; *p* < 0.001). A significant GAA × energy interaction was observed for breast yield (*p* < 0.001), where the highest breast yield was observed in birds receiving 1.2 g/kg GAA with the reduced-energy diet (24.54%), which was significantly greater than that of the standard-energy control group (22.62%; *p* < 0.001). For thigh yield, no significant interaction was detected (*p* = 0.98).

GAA supplementation significantly affected relative organ weights. Liver weight increased progressively with GAA level (*p* < 0.001). Gizzard weight decreased significantly with GAA supplementation (*p* < 0.001). Intestine weight was significantly reduced by the highest GAA level compared to control (*p* < 0.001). Dietary energy reduction significantly increased gizzard weight (*p* < 0.001) but had no significant effect on liver weight (*p* = 0.53) or intestine weight (*p* = 0.05). Significant GAA × energy interactions were observed for liver weight (*p* < 0.001), gizzard weight (*p* < 0.001), and intestine weight (*p* < 0.001).

### 6.4. Heat Stress Response

No significant main effect of GAA was observed for panting frequency (*p* = 0.38; Table 6). However, a significant GAA × energy interaction was detected (*p* < 0.001), indicating that the effect of GAA on panting frequency was context-dependent and varied with dietary energy level. The highest panting frequency was recorded in birds fed 1.2 g/kg GAA with the standard-energy diet (126.00 breaths/min), followed by the control group fed the reduced-energy diet (125.00 breaths/min). In contrast, the lowest panting frequency was observed in the standard-energy control group (101.00 breaths/min), followed by birds receiving 1.2 g/kg GAA with the reduced-energy diet (103.00 breaths/min). Notably, GAA supplementation at 1.2 g/kg reduced panting frequency in birds fed the reduced-energy diet, from 125.00 to 103.00 breaths/min (*p* < 0.001), indicating a substantial mitigation of heat stress under reduced-energy conditions. Conversely, under standard-energy conditions, 1.2 g/kg GAA increased panting frequency compared with the control treatment, from 101.00 to 126.00 breaths/min (*p* < 0.001), suggesting a potential increase in metabolic heat production when dietary energy was not limiting.

## 7. Discussion

The present study was conducted to evaluate the effects of graded levels of GAA (0, 0.6, and 1.2 g/kg) under standard and reduced (−100 kcal/kg) dietary energy on broiler performance, meat quality, carcass traits, and heat stress resilience. We tested five primary hypotheses: (i) GAA would improve growth performance and feed efficiency in a dose-dependent manner; (ii) meat quality parameters, particularly WHC and color, would be enhanced through improved post mortem energy metabolism; (iii) GAA would exhibit an energy-sparing effect, maintained growth performance comparable to that of birds fed standard-energy diets; (iv) GAA would mitigate heat-stress-induced physiological responses, as indicated by reduced panting frequency; and (v) significant GAA × energy interactions would exist, with the magnitude of GAA’s benefits dependent on dietary energy level. The following discussion addresses each hypothesis in light of our findings and the recent literature.

The significant improvements in final body weight, weight gain, and FCR observed with GAA supplementation in this study align with a substantial body of literature demonstrating the growth-promoting effects of GAA supplementation as a nutritional strategy in broiler chickens. GAA supplementation at 1.2 g/kg shows promise as a nutritional strategy for broiler production; however, further research in commercial settings is warranted to validate these findings. The dose-dependent response, with the greatest improvements at 1.2 g/kg GAA, is consistent with the findings of Ale Saheb Fosoul et al. [19], who reported that supplementation with 1.2 g/kg GAA improved broiler growth performance and helped offset the adverse effects of reduced dietary energy. Similarly, a recent study by Yen et al. [24] demonstrated that GAA supplementation at 1.2 g/kg feed achieved the lowest overall FCR (1.33) in Ross 308 broilers, supporting the pattern observed in the present study, where FCR improved from 1.51 in the control group to 1.43 in the 1.2 g/kg GAA group. The underlying mechanism for these improvements relates to the role of GAA as a precursor for creatine synthesis. As reviewed by Portocarero and Braun [25], GAA is a natural amino acid derivative that serves as the direct precursor of creatine synthesis in the animal body, possessing multiple physiological functions including regulating muscle energy metabolism, promoting muscle growth and development, and sparing arginine.

One of the most significant findings of this study is the large compensation of the reduced-energy diet by GAA supplementation at 1.2 g/kg. Birds receiving this combination achieved a final body weight comparable to that of those fed standard-energy diets with GAA and superior to control birds fed standard-energy diets without GAA. This finding is consistent with recent research on the energy-sparing effects of GAA. Paz et al. [26] evaluated the hepatic metabolism and performance of broilers fed reduced-energy diets, 50 kcal/kg less, with or without GAA supplementation, and found that birds fed GAA-supplemented diets showed improved performance compared to the unsupplemented reduced-energy group. Similarly, Pirgozliev et al. [12] demonstrated that supplementation with 0.06% GAA, equivalent to 600 g/t feed, more than compensated for performance depressions induced by reductions in dietary AMEn of approximately 50 to 100 kcal/kg. Although the magnitude of response may vary among studies, the present study observed clearer effects, which may be attributable to differences in basal diet composition, environmental conditions, or the longer duration of energy restriction.

The significant reduction in both drip loss (42.9%) and cooking loss (16.1%) at 1.2 g/kg GAA represents a substantial improvement in meat quality, as WHC is a critical determinant of meat juiciness, texture, and economic value. These findings are strongly supported by the recent literature. Dayan et al. [27] demonstrated that dietary inclusion of 0.06% GAA significantly reduced drip loss from 2.48% to 1.78% in broiler breast meat, while also reducing the risk of spaghetti meat myopathy by 30%. The reduction in drip loss observed in their study (approximately 28%) is consistent with the magnitude of improvement seen in the present study. Creatine loading pre-slaughter slows post mortem glycolysis and pH decline, reducing protein denaturation and preserving water-binding capacity [17]. GAA may also enhance antioxidant capacity and reduce lipid oxidation [28], suggesting that improved oxidative stability may have contributed to the observed reduction in yellowness (b* value). The darker, redder meat from GAA-supplemented birds is mechanistically consistent with the findings of Xu et al. [29], who reported that enhanced muscle creatine loading increased a* values and reduced the hue angle during refrigerated storage. The negative impact of energy restriction on meat quality likely relates to altered muscle glycogen stores and increased oxidative stress, partially mitigated by GAA as evidenced by the significant interaction for cooking loss.

The significant improvements in dressing percentage (5.2% increase), breast yield (14.8% increase), and thigh yield (12.8% increase) with GAA supplementation at 1.2 g/kg represent major economic benefits for broiler producers. These findings are consistent with those of Westreicher-Kristen et al. [30], who reported that breast yield was increased in GAA-supplemented groups compared to controls. Similarly, Lemme et al. [31] and Michiels et al. [8] reported increased breast meat yield with GAA. The mechanism involves creatine’s role in muscle cell differentiation and fusion [32], promoting protein deposition. However, their study found that breast meat redness (a*) decreased with GAA supplementation, which contrasts with the present findings, where the a* value increased by 45%. This discrepancy may be due to differences in the basal diet composition (particularly arginine-to-lysine ratios), duration of supplementation, or the specific GAA inclusion levels used. The reduction in gizzard and intestine weights observed in the present study suggests alterations in gastrointestinal development, but the underlying physiological mechanism remains unclear. This finding is supported by a recent study by Abudabos et al. [33], who reported that GAA supplementation notably improved intestinal morphometry in broilers, particularly in male birds. While this finding may indicate improved feed utilization efficiency, as has been suggested by previous studies [34,35], it should be noted that direct measurements of nutrient digestibility, digestive enzyme activity, or intestinal histomorphology were not performed in this study. Therefore, these findings warrant further investigation.

The complex interaction between GAA supplementation and dietary energy level on panting frequency reveals important considerations for nutritional management in hot climates. The 17.6% reduction in panting frequency observed with 1.2 g/kg GAA under reduced-energy conditions, compared with unsupplemented reduced-energy controls, suggests that GAA may exert thermoprotective effects when dietary energy is limited. However, the observation that GAA supplementation increased panting frequency in birds fed standard-energy diets (126.00 vs. 101.00 breaths/min) suggests that when energy is not limiting, GAA may increase metabolic rate and endogenous heat production. This finding highlights the context-dependent nature of GAA’s effects on thermoregulation. The practical implication is that GAA’s thermoprotective effects may be optimized when combined with moderate energy reduction, enabling birds to maintain performance while reducing metabolic heat load.

This is supported by the findings of Paz et al. [26], who reported that birds fed GAA-supplemented diets showed higher levels of very-low-density lipoproteins and triglycerides, indicating enhanced lipid metabolism that may have contributed to increased metabolic heat production. The practical implication is that GAA’s thermoprotective effects may be optimized when combined with moderate energy reduction, enabling birds to maintain performance while reducing metabolic heat load. This interpretation is further supported by Majdeddin et al. [14], who reported improved FCR and survival in broilers supplemented with GAA under cyclic heat stress. In addition, Li et al. [15] reported that 0.6 g/kg GAA reduced corticosterone levels and improved immune function under chronic heat stress.

It should be noted that heat stress was applied from day 27 to day 35, and the growth performance data presented in Table 3 cover the entire 35-day period, potentially confounding the effects of GAA on energy utilization and heat stress mitigation. While GAA’s benefits on growth performance likely reflect both improved energy metabolism and heat stress tolerance, we cannot definitively separate these effects with the current dataset. Future studies with segmented growth periods (pre- and post-heat-stress) would help distinguish between GAA’s energy-sparing effects and its potential thermoprotective effects.

## 8. Conclusions

Dietary GAA supplementation at 1.2 g/kg improved growth performance, feed efficiency, meat quality (WHC and color), and carcass yield (dressing percentage, breast and thigh meat) in broiler chickens, while reducing gizzard and intestine weights, suggesting potential alterations in gastrointestinal development. Importantly, GAA supplementation largely compensated for a 100 kcal/kg reduction in dietary energy, as evidenced by comparable body weights to standard-energy control groups, supporting its potential role in cost-effective diet formulation. Under cyclic heat stress, the combination of 1.2 g/kg GAA with reduced dietary energy decreased panting frequency, indicating GAA demonstrated context-dependent effects on panting frequency, with benefits observed primarily under reduced-energy conditions. However, under standard-energy conditions, GAA increased panting frequency, indicating that the effects of GAA on thermoregulation depend on energy availability and should be carefully considered in heat stress management strategies. Overall, GAA supplementation at 1.2 g/kg supports the potential of GAA supplementation as a nutritional strategy to support sustainable and resilient broiler production, particularly in hot-climate regions and under conditions of volatile feed prices.

Some limitations should be acknowledged. While panting frequency is a well-established indicator of heat stress response, this study did not directly measure other physiological stress markers such as corticosterone, HSP70, rectal temperature, or oxidative stress parameters. Future studies should include such measurements to provide a more comprehensive assessment of heat stress tolerance. The interpretation of reduced gizzard and intestinal weights as indicators of altered digestive efficiency remains unclear, as direct measurements of nutrient digestibility, AME, digestive enzyme activity, or intestinal histomorphology were not performed. The energy compensation should be interpreted cautiously, as “complete compensation” could not be statistically proven; rather, the data demonstrate that growth performance was maintained comparable to standard-energy controls. Finally, the heat stress protocol (8 h/day for 8 days) represents cyclic rather than acute heat stress, and the effects of GAA under acute heat stress conditions at the farm scale warrant further investigation.

## Figures and Tables

**Table 1:** Experimental layout.

Sr. No.	Treatment Description	Experimental Design
1	Basal diet + standard energy	Treatments = 6
2	Basal diet − 100 kcal/kg energy	Replicates per treatment = 6
3	Basal diet + 0.6 g/kg GAA + standard energy	Experimental units/pens = 6 × 6 = 36
4	Basal diet + 0.6 g/kg GAA − 100 kcal/kg energy	Birds per replicate = 10
5	Basal diet + 1.2 g/kg GAA + standard energy	Total birds = 6 × 6 × 10 = 360
6	Basal diet + 1.2 g/kg GAA − 100 kcal/kg energy	Strain: Ross 308; design: CRD

GAA = guanidinoacetic acid; CRD = completely randomized design.

**Table 2:** Ingredient composition and nutrient contents of the basal diets.

Ingredients (%)	Starter SE	Starter LE	Grower SE	Grower LE	Finisher SE	Finisher LE
Corn	32	27	29.28	29.8	23.2	25.05
Rice tips	20	20	20	20	30	30
Wheat	0	0	10	10	10	10
Soybean meal	30	30	16	16	13	13
Canola meal	11	11	10	10	11	11
Meat and bone meal	4	4	2	2	4	4
Poultry byproduct meal	0	0	4	4	4	4
Poultry oil	0	0	1.5	1	1.85	0
Guar meal	0	0	2	2	0	0
Rapeseed meal	0	0	2	2	0	0
Wheat bran	0	5	0	0	0	0
Lysine sulphate	0.53	0.49	0.8	0.79	0.82	0.82
DL-methionine	0.38	0.37	0.33	0.32	0.29	0.29
Isoleucine	0.06	0.05	0.16	0.16	0.14	0.14
Valine	0.05	0.04	0.11	0.10	0.08	0.08
Threonine	0.18	0.17	0.21	0.20	0.21	0.21
Arginine	0.03	0.02	0.14	0.13	0.18	0.18
Salt	0.16	0.16	0.17	0.16	0.12	0.12
Soda bicarb	0.19	0.19	0.16	0.19	0.10	0.10
Dicalcium phosphate	0.23	0.23	0.05	0.23	0.05	0.23
Limestone chips	0.7	0.7	0.66	0.7	0.4	0.4
Mineral premix *	0.05	0.05	0.05	0.05	0.05	0.05
Vitamin premix *	0.05	0.05	0.05	0.05	0.05	0.05
Nutrient Contents						
Metabolizable energy (kcal/kg)	2920	2820	3060	2960	3150	3050
Crude fat (%)	3.45	3.2	4.8	3.65	5.4	3.85
Crude fiber (%)	3.75	4.1	3.9	4.05	3.8	3.8
Crude protein (%)	22.8	22.8	21.3	21.3	19.9	19.9
Digestible lysine (%)	1.28	1.25	1.15	1.12	1.05	1.05
Digestible methionine (%)	0.65	0.64	0.58	0.56	0.52	0.52
Digestible arginine (%)	1.45	1.42	1.25	1.22	1.18	1.18
Calcium (%)	0.95	0.95	0.88	0.88	0.8	0.8
Available Phosphorus (%)	0.45	0.45	0.42	0.42	0.4	0.4

SE, standard energy; LE, low energy (−100 Kcal/kg). * Added per kg of diet.

**Table 3:** Effect of GAA and energy level on broiler growth performance (1–35 days).

Treatment	Final Body Weight (g)	Body Weight Gain (g)	Feed Intake (g)	FCR
GAA				
0	2006.67 ^c^	1963.50 ^c^	2974.33 ^b^	1.51 ^c^
0.6	2081.50 ^b^	2038.50 ^b^	3018.83 ^a^	1.48 ^b^
1.2	2144.50 ^a^	2101.67 ^a^	3008.33 ^a^	1.43 ^a^
SEM	12.40	12.40	8.76	0.021
Energy				
Standard	2099.04 ^a^	2056.04 ^a^	3014.04 ^a^	1.46 ^a^
−100 kcal	2055.00 ^b^	2012.00 ^b^	2986.00 ^b^	1.48 ^b^
SEM	10.20	10.20	6.14	0.015
Interaction				
0 × Standard	2050.55 ^c^	2007.55 ^c^	2999.55	1.49 ^b^
0 × −100 kcal	1962.50 ^d^	1919.50 ^d^	2949.50	1.53 ^c^
0.6 × Standard	2093.02 ^b^	2050.02 ^b^	3029.02	1.47 ^b^
0.6 × −100 kcal	2070.65 ^bc^	2027.65 ^bc^	3008.65	1.48 ^b^
1.2 × Standard	2155.59 ^a^	2112.59 ^a^	3014.59	1.42 ^a^
1.2 × −100 kcal	2134.53 ^a^	2091.53 ^a^	3001.53	1.43 ^a^
SEM	14.80	14.80	10.72	0.026
ANOVA		Probability		
GAA	<0.01	<0.01	<0.01	<0.01
Energy	<0.01	<0.01	<0.01	<0.01
GAA × Energy	<0.01	<0.01	0.45	<0.01

For variables with non-significant GAA × energy interactions (feed intake, *p* = 0.45), interaction cell means are presented without superscript letters. For variables with significant interactions, superscript letters indicate significant differences between interaction cell means. Values are means (*n* = 6 replicates per treatment). ^a–d^ Means within a column and within each factor (GAA and energy) or interaction (where significant) with different superscript letters differ significantly (*p* < 0.05; two-way ANOVA with Tukey’s post hoc test). Abbreviations: GAA, guanidinoacetic acid; FCR, feed conversion ratio; SEM, standard error of the mean.

**Table 4:** Effect of GAA and energy level on broiler meat quality at 35 days.

Items	Drip Loss (%)	Cooking Loss (%)	Meat Color (15 min)		
			L*	a*	b*
Main Effects—GAA					
0 g/kg GAA	3.75 ^a^	23.82 ^a^	55.70 ^a^	4.18 ^c^	9.67 ^a^
0.6 g/kg GAA	2.98 ^b^	22.12 ^b^	53.85 ^b^	5.25 ^b^	8.62 ^b^
1.2 g/kg GAA	2.14 ^c^	19.99 ^c^	52.35 ^c^	6.06 ^a^	7.43 ^c^
SEM	0.18	0.45	0.42	0.18	0.22
Main Effects—Energy					
Standard Energy	2.58 ^b^	20.88 ^b^	52.95 ^b^	5.60 ^a^	8.00 ^b^
−100 kcal/kg	3.34 ^a^	23.08 ^a^	54.97 ^a^	4.72 ^b^	9.15 ^a^
SEM	0.15	0.38	0.35	0.15	0.18
Interactions (GAA × Energy)					
0 g/kg GAA × Standard	3.34	22.48 ^b^	54.80	4.58	9.10
0 g/kg GAA × −100 kcal/kg	4.17	25.17 ^a^	56.60	3.78	10.25
0.6 g/kg GAA × Standard	2.60	21.28 ^c^	52.76	5.65	8.11
0.6 g/kg GAA × −100 kcal/kg	3.36	22.98 ^b^	54.93	4.85	9.13
1.2 g/kg GAA × Standard	1.79	18.89 ^d^	51.28	6.58	6.78
1.2 g/kg GAA × −100 kcal/kg	2.50	21.09 ^c^	53.44	5.55	8.08
SEM	0.22	0.55	0.51	0.22	0.27
ANOVA		Probability			
GAA	<0.01	<0.01	<0.01	<0.01	<0.01
Energy	<0.01	<0.01	<0.01	<0.01	<0.01
GAA × Energy	0.45	<0.01	0.39	0.19	0.20

For variables with non-significant GAA × energy interactions (drip loss, L*, a*, and b*), interaction cell means are presented without superscript letters. Only significant interactions (cooking loss) retain superscripts. Values are means (*n* = 6 replicates per treatment; 2 birds per replicate). ^a–d^ Means within a column and within each factor (GAA and energy) with different superscript letters differ significantly (*p* < 0.05; two-way ANOVA with Tukey’s post hoc test). Abbreviations: GAA, guanidinoacetic acid; L*, lightness; a*, redness; b*, yellowness; SEM, standard error of the mean.

**Table 5:** Effect of GAA and energy level on broiler carcass characteristics at 35 days.

Items	Live BW	Dressing	Breast Yield	Thigh Yield	Liver	Gizzard	Intestine
	(g)	(%)	(%)	(%)	(%)	(%)	(%)
Main Effects—GAA							
0 g/kg GAA	1990.11 ^b^	71.19 ^c^	21.83 ^c^	16.74 ^c^	2.57 ^c^	1.95 ^a^	5.90 ^a^
0.6 g/kg GAA	2011.33 ^b^	73.07 ^b^	23.26 ^b^	17.66 ^b^	2.72 ^b^	1.86 ^b^	5.81 ^a^
1.2 g/kg GAA	2105.58 ^a^	74.89 ^a^	25.06 ^a^	18.88 ^a^	2.79 ^a^	1.71 ^c^	5.50 ^b^
SEM	13.80	0.42	0.31	0.22	0.04	0.03	0.05
Main Effects—Energy							
Standard Energy	2070.61 ^a^	73.92 ^a^	24.06 ^a^	18.07 ^a^	2.70 ^a^	1.77 ^b^	5.70 ^a^
−100 kcal/kg	2001.23 ^b^	72.18 ^b^	22.09 ^b^	17.45 ^b^	2.69 ^a^	1.91 ^a^	5.77 ^a^
SEM	11.40	0.35	0.26	0.18	0.03	0.02	0.04
Interactions (GAA × Energy)							
0 g/kg GAA × Standard	2024.34 ^b^	72.17	22.62 ^d^	17.05	2.57 ^c^	1.88 ^c^	5.88 ^a^
0 g/kg GAA × −100 kcal/kg	1955.33 ^c^	70.21	21.05 ^e^	16.43	2.58 ^c^	2.02 ^a^	5.91 ^a^
0.6 g/kg GAA × Standard	2073.17 ^a^	73.93	24.03 ^c^	17.96	2.68 ^b^	1.77 ^d^	5.69 ^b^
0.6 g/kg GAA × −100 kcal/kg	1949.18 ^c^	72.21	22.48 ^d^	17.35	2.75 ^b^	1.96 ^b^	5.92 ^a^
1.2 g/kg GAA × Standard	2112.13 ^a^	75.67	25.52 ^a^	19.20	2.84 ^a^	1.66 ^e^	5.53 ^bc^
1.2 g/kg GAA × −100 kcal/kg	2099.12 ^a^	74.11	24.54 ^b^	18.57	2.74 ^a^	1.75 ^d^	5.46 ^c^
SEM	16.80	0.51	0.38	0.27	0.05	0.04	0.06
ANOVA			Probability				
GAA	<0.01	<0.01	<0.01	<0.01	<0.01	<0.01	<0.01
Energy	<0.01	<0.01	<0.01	<0.01	0.53	<0.01	0.05
GAA × Energy	<0.01	0.15	<0.01	0.98	<0.01	<0.01	<0.01

For variables with non-significant GAA × energy interactions (dressing percentage, *p* = 0.15; thigh yield, *p* = 0.98), interaction cell means are presented without superscript letters. For variables with significant interactions, superscript letters indicate significant differences between interaction cell means. Values are means (*n* = 6 replicates per treatment; 2 birds per replicate). ^a–e^ Means within a column and within each factor (GAA and energy) or interaction (where significant) with different superscript letters differ significantly (*p* < 0.05; two-way ANOVA with Tukey’s post hoc test). Abbreviations: GAA, guanidinoacetic acid; SEM, standard error of the mean; Live BW, live body weight.

**Table 6:** Effect of GAA and energy level on panting frequency under cyclic heat stress.

Items	Panting Frequency (Breaths/min)
Main Effects—GAA	
0 g/kg GAA	116.00 ^a^
0.6 g/kg GAA	115.25 ^a^
1.2 g/kg GAA	115.16 ^a^
SEM	1.46
Main Effects—Energy	
Standard Energy	116.61 ^a^
−100 kcal/kg	114.33 ^b^
SEM	1.54
Interactions (GAA × Energy)	
0 g/kg GAA × Standard	101.00 ^c^
0 g/kg GAA × −100 kcal/kg	125.00 ^a^
0.6 g/kg GAA × Standard	116.00 ^b^
0.6 g/kg GAA × −100 kcal/kg	114.50 ^b^
1.2 g/kg GAA × Standard	126.00 ^a^
1.2 g/kg GAA × −100 kcal/kg	103.00 ^d^
SEM	1.24
ANOVA	Probability
GAA	0.38
Energy	<0.01
GAA × Energy	<0.01

Values are means ± SEM (*n* = 6 replicates per treatment; 2 birds per replicate). ^a–d^ Means within a column and within each factor (GAA, energy, and GAA × energy) with different superscript letters differ significantly (*p* < 0.05; two-way ANOVA with Tukey’s post hoc test). Abbreviations: GAA, guanidinoacetic acid; SEM, standard error of the mean.

## Data Availability

The data presented in this study are available upon reasonable request from the corresponding author.
